# The Tumor Microenvironment Factors That Promote Resistance to Immune Checkpoint Blockade Therapy

**DOI:** 10.3389/fonc.2021.641428

**Published:** 2021-06-29

**Authors:** Bonnie L. Russell, Selisha A. Sooklal, Sibusiso T. Malindisa, Lembelani Jonathan Daka, Monde Ntwasa

**Affiliations:** ^1^ Department of Life & Consumer Sciences, University of South Africa, Johannesburg, South Africa; ^2^ Innovation Hub, Buboo (Pty) Ltd, Pretoria, South Africa

**Keywords:** PD-1, CTLA-4, Immune checkpoint inhibitor, resistance, tumor microenvironment

## Abstract

Through genetic and epigenetic alterations, cancer cells present the immune system with a diversity of antigens or neoantigens, which the organism must distinguish from self. The immune system responds to neoantigens by activating naïve T cells, which mount an anticancer cytotoxic response. T cell activation begins when the T cell receptor (TCR) interacts with the antigen, which is displayed by the major histocompatibility complex (MHC) on antigen-presenting cells (APCs). Subsequently, accessory stimulatory or inhibitory molecules transduce a secondary signal in concert with the TCR/antigen mediated stimulus. These molecules serve to modulate the activation signal’s strength at the immune synapse. Therefore, the activation signal’s optimum amplitude is maintained by a balance between the costimulatory and inhibitory signals. This system comprises the so-called immune checkpoints such as the programmed cell death (PD-1) and Cytotoxic T lymphocyte-associated antigen-4 (CTLA-4) and is crucial for the maintenance of self-tolerance. Cancers often evade the intrinsic anti-tumor activity present in normal physiology primarily by the downregulation of T cell activation. The blockade of the immune checkpoint inhibitors using specific monoclonal antibodies has emerged as a potentially powerful anticancer therapy strategy. Several drugs have been approved mainly for solid tumors. However, it has emerged that there are innate and acquired mechanisms by which resistance is developed against these therapies. Some of these are tumor-intrinsic mechanisms, while others are tumor-extrinsic whereby the microenvironment may have innate or acquired resistance to checkpoint inhibitors. This review article will examine mechanisms by which resistance is mounted against immune checkpoint inhibitors focussing on anti-CTL4-A and anti-PD-1/PD-Ll since drugs targeting these checkpoints are the most developed.

## Introduction

Cancers often evade the intrinsic anti-tumor activity present in normal physiology through various mechanisms one of which is the downregulation of T cell activation. Through genetic and epigenetic alterations, cancer cells present the immune system with a diversity of antigens, which are distinguishable from self. Antigen-specific T cell activation is initiated by a signal mediated by the interaction of the T cell receptor (TCR) with an antigen that is bound to the major histocompatibility complex (MHC) on antigen presenting cells and another signal transduced through co-stimulatory molecules belonging to the B7 family. The optimum amplitude of activation signal is maintained by a balance between this costimulatory signal and an inhibitory one also mediated by the B7 family (1). This system comprises the so-called immune checkpoints mediated by the inhibitory molecules and is crucial for the maintenance of self-tolerance. The blockade of the immune checkpoint inhibitors has emerged as a potentially powerful strategy for anti-cancer therapy and several drugs, mainly for solid tumors, have been approved **(** **Table 1** **)** (35, 36).

**Table 1:** List of FDA-approved Immune Checkpoint Inhibitors (ICIs) targeting CTLA-4, PD-1 and PD-L1.

Drug (Trade name)	Company	Date of approval	Indication	References
***CTLA-4 inhibitors***				
Ipilimumab (Yervoy^®^)	Bristol-Myers Squibb	2011	Melanoma	(2)
			colorectal cancer	(3)
			Renal cell carcinoma	(4)
***PD-1 inhibitors***				
Nivolumab (Opdivo^®^)	Bristol-Myers Squibb	2014	Melanoma	(5)
			Hodgkin’s lymphoma	(6)
			Diffuse large B-cell lymphoma	(7)
			Urothelial cancer	(8)
			Colorectal cancer	(3)
			Hepatocellular carcinoma	(9)
			Non-small cell lung cancer	(10)
			Small cell lung cancer	(11)
			Renal cell carcinoma	(12)
			Squamous cell carcinoma	(13)
Pembrolizumab (Keytruda^®^)	Merck	2014	Melanoma	(14)
			Cervical cancer	(15)
			Hodgkin’s lymphoma	(16)
			Diffuse large B-cell lymphoma	(17)
			Gastric cancer	(18)
			Urothelial cancer	(19)
			Colorectal cancer	(20)
			Hepatocellular carcinoma	(21)
			Non-small cell lung cancer	(22)
			Small cell lung cancer	(23)
			Renal cell carcinoma	(24)
			Squamous cell carcinoma	(25)
			Esophageal cancer	(26)
			Merkel cell carcinoma	(27)
Cemiplimab (Libtayo^®^)	Sanofi	2018	Cutaneous squamous cell carcinoma	(28)
***PD-L1 inhibitors***				
Atezolizumab (Tecentriq^®^)	Roche, Genentech	2016	Non-small cell lung cancer	(29)
			Triple negative breast cancer	
Avelumab (Bavencio^®^)	Merck, Pfizer	2017	Merkel cell carcinoma	(30)
			Renal cell carcinoma	(31)
			Urothelial cancer	(32)
Durvalumab (Imfinzi^®^)	AstraZeneca	2017	Bladder cancer	(33)
			Non-small cell lung cancer	(34)

Cancers develop within a diverse and dynamic microenvironment and possess mechanisms to survive unfavourable physiological machinery designed to suppress carcinogenesis. Thus, they are equipped with strategies to reprogram the microenvironment metabolically and immunologically. For example, cancers develop mechanisms to switch off the physiological immune response by blocking activated T cells to protect themselves from cytotoxic killing. Thus, during cancer progression, the immune checkpoint pathways mediated by the structurally similar co-inhibitory receptors; Programmed cell Death 1 (PD-1) and the Cytotoxic T lymphocyte-associated antigen-4 (CTLA-4) or CD152 receptors are often usurped by cancer cells to evade immune surveillance. These two receptors, which form part of a growing list of checkpoint inhibitors, are the foremost targets for immune checkpoint inhibition-based drug development in recent years **(** **Table 1** **)**.

In this review we examine the mechanisms of inhibitors targeting the immune checkpoint pathways PD-1/PD-L1 and CTLA-4, as well as the types of resistance that can develop against them.

## CTLA-4 and PD-1 Immune Checkpoint Signalling Pathways

The CTLA-4, which is the first co-inhibitory immune checkpoint receptor to be identified, is constitutively expressed on both CD4+ and CD8+ T lymphocytes (37). CD28 and CTLA-4 are both capable of binding two important ligands, namely B7.1 (also known as CD80) and B7.2 (also known as CD86) (38). CTLA-4 expression is up regulated in T cells after activation. This is particularly significant in cancer cells as CTLA-4 has a higher binding affinity to both ligands, compared to CD28. Consequently, it is plausible that the role of the CTLA-4 expressed on the surface of T cells is to decrease T cell activation by competing with CD28 for ligand binding as well as active removal of B7.1 and B7.2 from the cell surface of antigen-presenting cells (APCs) (39). It counteracts the activity of the co-stimulatory CD28 upon TCR engagement by the antigen-MHC complex on APCs (40). Upon T cell activation CTLA-4 is translocated *via* a genetically programed pathway to the cell surface where it competes for binding with CD28. At the cell surface CTLA-4 is stabilized by src kinase-mediated phosphorylation and binds with higher affinity to B7 ligands when compared with CD28. Intracellularly CTLA-4 transduces signals *via* PP2A and PI3K (41).

**Figure 1:**
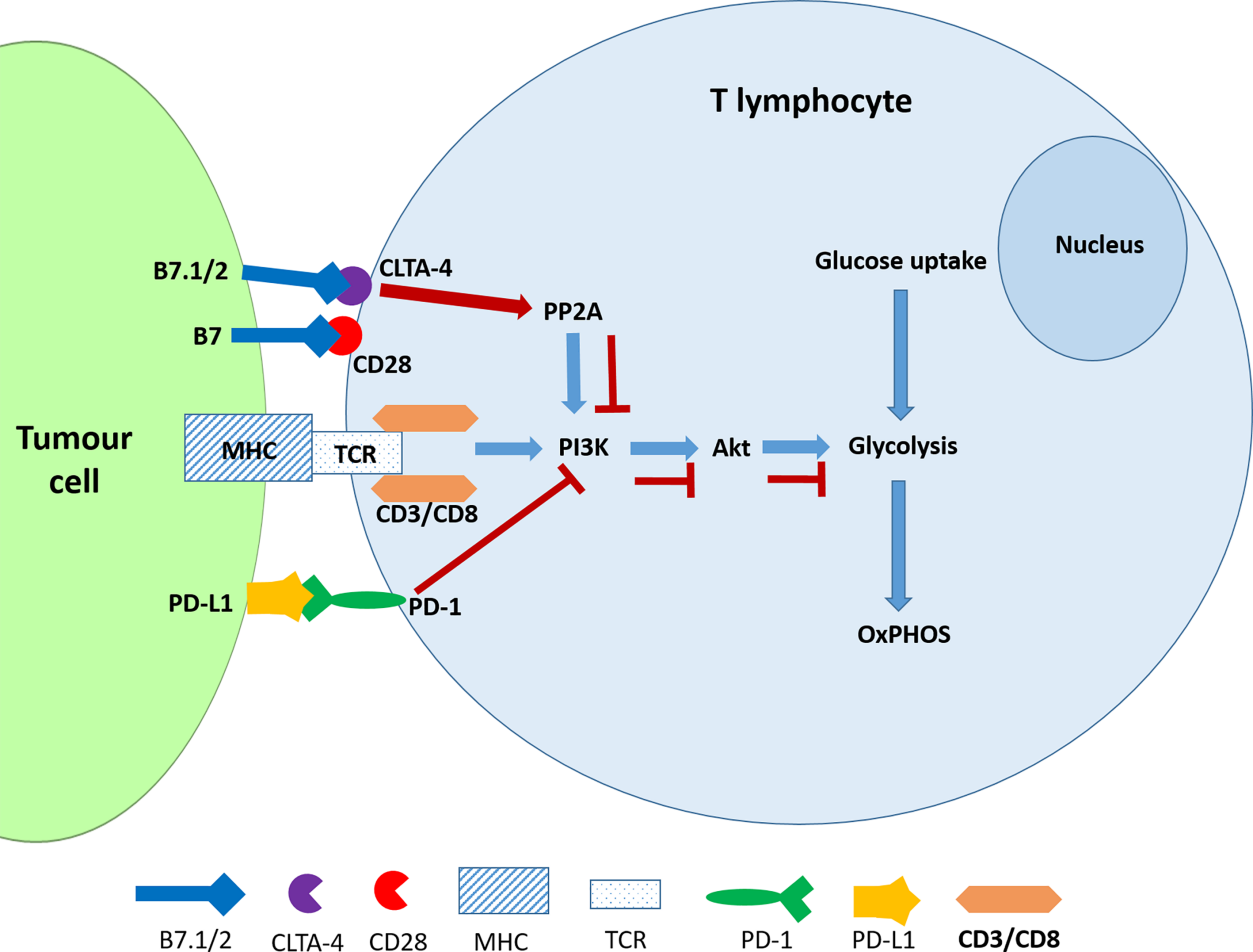
CTLA-4 and PDL-1 ligation interferes with glucose metabolism in activated T cells. The ligation of PD-1 blocks the activation of PI3K and consequently the Akt signalling pathway resulting the inhibition of glycolysis. CTLA-4 accomplishes the same outcome by activating the phosphatase PP2A.

PD-1 is an inhibitor of both adaptive and innate immune responses and is more broadly expressed than CTLA-4 on activated T cells, B cells and myeloid cells and its depletion in experimental mice results in the disruption of immune tolerance and in multiple autoimmune features (42, 43). The TCR transduces the signal *via* the PI3K/Akt pathway and positively regulates glucose metabolism, which is reprogrammed during T cell activation **(** **Figure 1** **)**. A negative signal during TCR activation may occur *via* a ligated PD-1 receptor, which mediates the recruitment of phosphatases, SHP2 (and/or SHP1) to dephosphorylate TCR-proximal molecules and displace the co-stimulatory molecule, CD28, thereby blocking lymphocyte activation. PD-1 ligation also directly inhibits phosphatidylinositol 4,5-isphosphate-3 kinase (PI3K) (44). In the absence of PD-1, TCR signalling leads to Akt activation thereby promoting key cellular activities including glucose metabolism, cytokine production and phosphorylated glycogen synthase kinase-3 (GSK-3β_P) associated events which include glycogen synthesis in the liver and in the muscles (45). Hence the inhibition of GSK-3 leads to the development of cancer and other developmental diseases (46). The ligands of PD-1 and CTLA-4 receptors belong to the B7 family and function by mediating “co-stimulatory” or “co-inhibitory” signals through the CD28 family of receptors on lymphocytes (47). Engagement of PD-1 by its ligands, PDL-1 and PDL-2, which are expressed on antigen presenting cells downregulates lymphocyte activation (48).

The evidence has shown that the CTLA-4 and PD-1 receptors may inhibit T-cell activation but use different signalling and synergistic pathways. Furthermore, the ligation of these receptors by their physiological ligands leads to the downregulation of glycolysis (45). It is noteworthy that, like cancer cells, activated T cells also exhibit the Warburg Effect or aerobic glycolysis which is characterised by elevated glycolysis and downregulated oxidative phosphorylation and is driven by mechanistic target of rapamycin (mTOR) signalling (49). The antagonistic effect of checkpoint inhibitors should therefore affect the metabolic reprogramming that would have occurred in activated T cells. However, this has not been specifically investigated according to our knowledge.

It has been shown that T cell activation requires upregulation of glucose metabolism and that while glucose deprivation does not affect proliferation, it diminishes the effector activities of T cells thereby driving cancer progression. Alternatively, when glycolysis was inhibited in CD8^+^ T cell using 2-deoxy-D-glucose (2-DG) in the mouse sarcoma model, interferon gamma (IFNγ) but not Interleukin-2 (IL-2) production was inhibited. Furthermore, a large-scale transcriptional analysis also showed that only 10% of genes induced by T cell activation were inhibited by 2-DG. This small subset of genes comprised those involved in effector functions (50). These observations suggest that the metabolic reprogramming associated with T cell activation specifies their functional properties However, the impact of glucose metabolic profiles of the tumor microenvironment components on immune checkpoint blockade therapy is still not well understood.

In the solid tumor microenvironment, competition for glucose between cancer cells and tumor infiltrating CD8+ lymphocytes has been shown to result in the suppression of the T cell metabolic phenotype and effector capacity. Furthermore, it was shown that the glycolytic phenotype of cancer cells suppresses the metabolic programme and effector activities of T cells (51). Importantly, this study showed that anti-CTLA-4 and anti-PD-1 antibodies could reverse the antagonistic impact exerted by cancer cells on the TME.

Another question that requires attention is the comparative attractiveness of these receptors as therapeutic targets. Phenotypic differences in *PD-1* and *CTLA-4* knock-out mice show distinct outcomes that reveal critical features that suggests different responses to therapies that target these receptors. PD-1^-/-^ mice spontaneously develop lupus-like glomerulonephritis and arthritis. This phenotype is accelerated and characterized with extensive lymphadenopathy when the Fas or lymphoproliferation (*lpr^-/-^*) mutation is added. On the other hand, transgenic mice with CLTA-4 deficiency rapidly develop lymphoproliferative disease, multi-organ lymphocytic infiltration severe myocarditis and pancreatitis. Moreover, this mutation is lethal within four weeks (52, 53). These observations indicate that the blockade of PD-1 might be less toxic when compared to CTLA-4.

## Immune Checkpoint Inhibitors

### Mechanism of Inhibitors Targeting CTLA-4

**Figure 2:**
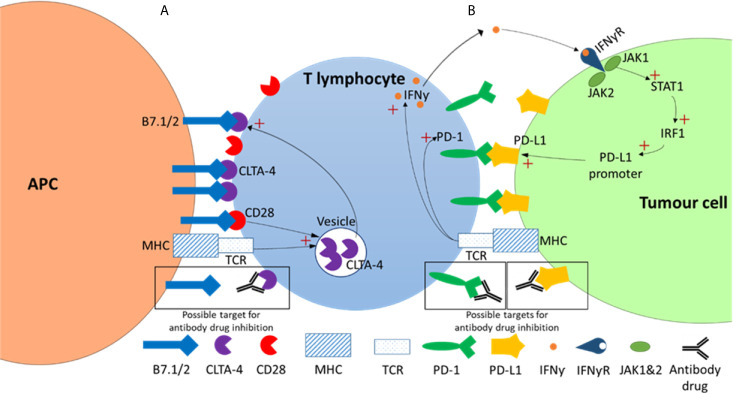
CTLA-4 and PD-1 checkpoint inhibitor pathways. **(A)** CTLA-4 pathway. In this pathway strong TCR-HMC and CD28-B7 binding signals initiate the exocytosis of the CTLA-4 from the intracellular vesicles to the T cell surface. As CLTA-4 has a higher binding affinity then CD28 for B7, this results in a net negative signal that results in reduced T cell proliferation, survival and a decrease in growth cytokines such as IL-2. **(B)** In the PD-1/PD-L1 pathway TCR-HMC signalling up regulates both PD-1 and interferon-​γ (IFNƴ) expression. The increased of IFNƴ in the tumor microenvironment activates the signalling pathway of Janus kinase (JAK)/signal transducer and activator of transcription (STAT) which activates the transcription factor interferon regulatory factor 1 (IRF1), which in turn induces PD-L1 expression. PD-1/PD-L1 interaction results in in a net negative signal and ultimately reduced T cell survival, proliferation and cytotoxic production. Possible antibody drug targets in both pathways are indicated showing antibody-target interaction (within black boxes).

CTLA-4 functions as a negative regulator of T-cell effector function and therefore presented as an attractive target for cancer therapy. Inhibitors targeting CTLA-4 act by preventing the binding between CTLA-4 (on T-cells) and B7 ligands (on APCs) (**Figure 2**). As a result, Treg-associated immune suppression is inhibited and T-cell effector function is promoted, allowing the immune system to mount a response (54–56). An influential clinical trial whereby improved survival rates were seen when patients with unresectable melanomas (stage III/IV) were treated with an anti-CTLA-4 monoclonal antibody ultimately led to the FDA approval of the first immune checkpoint inhibitor, ipilimumab, for cancer therapy (2).

Ipilimumab, marketed as Yervoy^®^ by Bristol-Myers Squibb, is a human IgG1κ anti-CTLA-4 monoclonal antibody. Ipilimumab was originally granted FDA approval for late stage, unresectable melanomas in 2011. It has subsequently been approved for patients with cutaneous melanoma, renal cell carcinoma and metastatic colorectal cancer as shown in **Table 1** (3, 4, 12, 57). Currently, ipilimumab remains as the only checkpoint inhibitor targeting CTLA-4.

### Mechanism of Inhibitors Targeting PD-1

The interaction between PD-1 (on T-cells) and its ligand, PD-L1 (on APCs) (**Figure 2**), has an inhibitory effect on T-cell effector activity. The PD-1/PD-L1 pathway therefore represents an additional negative regulator of immune responses and a key mechanism in tumor evasion (58). Inhibitors that target PD-1 act by preventing its binding to PD-L1 (**Figure 2**). This interferes with the feedback mechanism between T-cells and tumor cells in the tumor microenvironment and consequently restores T-cell effector function enhancing anti-tumor activity (36).

Following the outcome of the CheckMate-037 trial, nivolumab received FDA approval in 2014 for the treatment of unresectable or metastatic melanoma in patients whose cancers had progressed following ipilimumab treatment ± BRAF inhibitors (2). Nivolumab is a human IgG4κ anti-PD-1 monoclonal antibody marketed as Opdivo^®^ by Bristol-Myers Squibb. Nivolumab represented the first immune checkpoint inhibitor targeting PD-1 to be granted FDA approval. Its approval was subsequently expanded for the treatment of various cancers including cervical cancer (59), gastric cancer (60), urothelial cancer (8), Hodgkin’s lymphoma (6), hepatocellular carcinoma (9), squamous cell carcinoma (13, 61), colorectal cancer (3), non-small cell lung cancer (10), diffuse large B-cell lymphoma (62), renal cell carcinoma (12) and small cell lung cancer (5, 6, 8–13, 61, 63) (**Table 1**).

In 2014 an additional PD-1 inhibitor, pembrolizumab, was granted accelerated approval as an alternative for nivolumab in patients with unresectable or metastatic melanoma based on the results from the NCT01295827 clinical trial (14, 64). Pembrolizumab, a humanized IgG4κ anti-PD-1 monoclonal antibody marketed as Keytruda^®^ by Merck, later received expanded approval for the treatment of various cancers including cervical cancer (15), endometrial carcinoma (65), esophageal cancer (26), gastric cancer (18), urothelial cancer (19), Hodgkin’s lymphoma (16), hepatocellular carcinoma (21), Merkel cell carcinoma (27), squamous cell carcinoma (25), colorectal cancer (20, 66), non-small cell lung cancer (22), diffuse large B-cell lymphoma (17), renal cell carcinoma (24) and small cell lung cancer (15–22, 24–27, 66, 67).

Cemiplimab, a human IgG4κ anti-PD-1 monoclonal antibody marketed as Libtayo^®^ by Sanofi, is the most recent immune checkpoint inhibitor to be given FDA approval. In 2018, cemiplimab was approved for the treatment of metastatic cutaneous squamous cell carcinoma (28).

### Mechanism of Inhibitors Targeting PD-L1

Similar to inhibitors targeting PD-1, PD-L1 inhibitors aim to disrupt the interaction between PD-1 and PD-L1 in the tumor microenvironment. Inhibiting PD-1/PD-L1 results in the stimulation of T-cell anti-tumor activity as described previously (36, 68).

The first PD-L1 inhibitor granted FDA approval was Atezolizumab in 2016. Atezolizumab is a human IgG1κ anti-PD-L1 monoclonal antibody marketed as Tecentriq^®^, by Genentech and Roche. The mAb was found to be effective for the treatment of metastatic urothelial carcinoma following platinum chemotherapy (69). The therapy was subsequently approved for treatment of metastatic non-small-cell lung carcinoma (NSCLC) (29) and advanced urothelial carcinoma in patients that are ineligible for chemotherapy (19). In 2018, Atezolizumab was further approved for the treatment of metastatic NSCLC in combination with chemotherapy and bevacizumab, a mAb targeting VEGF (70). Following the first combinational therapy, Atezolizumab was subsequently approved in combination with paclixatel (71) and chemotherapy (72) for the treatment of metastatic triple negative breast cancer (TNBC) and small cell lung cancer (SCLC), respectively.

Avelumab, marketed as Bavencio^®^ by Merck/Pfizer, is a human IgG1λ monoclonal antibody that targets PD-L1. Avelumab was first approved by the FDA for the treatment of Merkel cell carcinoma in 2017 (30). Following its first approval, avelumab was granted further approval for the treatment of locally advanced and metastatic urothelial carcinoma (32). In 2019, avelumab was approved for the treatment of advanced renal cell carcinoma (RCC) in combination with axitinib, a tyrosine kinase inhibitor (31).

Another PD-L1 inhibitor, durvalumab, was granted FDA approval in 2017 for the treatment of advanced bladder cancer in patients that previously did not respond to chemotherapy or ineligible for the treatment (33). Durvalumab is a humanized IgG1κ anti-PD-L1 monoclonal antibody marketed as Imfinzi^®^ by AstraZeneca. In 2019, the immune checkpoint inhibitor was approved for the treatment of unresectable stage III NSCLC (34).

### The Mechanism of Next Generation Inhibitors Targeting LAG-3, TIM-3, TIGIT, VISTA and B7-H3

CTLA-4, PD-1 and PD-L1 are the most broadly studied checkpoints. However, given the success seen with previous checkpoint inhibitors, new inhibitory pathways and next generation inhibitors targeting LAG-3, TIM-3, TIGIT, VISTA and B7-H3 are being investigated. The mechanisms of these checkpoints as well as inhibitors that are currently in clinical trials will be described briefly.

Lymphocyte activation gene-3 (LAG-3 or CD223) is a membrane receptor constitutively expressed by T cells and natural killer cells. LAG-3 interacts with MHC class II resulting in a negative regulatory effect over T cell function (73). This interaction normally prevents tissue damage and autoimmunity, however, tumor-infiltrating lymphocytes (TILs) found in the TME upregulates LAG-3 thereby promoting cell dysfunction, immune exhaustion and favorable conditions for tumor growth (74). Thus, disrupting the LAG-3/MCH II interaction with blockade therapy should encourage immune activation and anti-tumor responses.

T cell immunoglobulin-3 (TIM-3) is an immune checkpoint expressed on numerous cells including effector T cells, B cells, Tregs, macrophages and natural killer cells (75). Its main ligand is galactine-9, but it is also known to interact with phosphatidyl serine and carcinoembryonic antigen-related cell adhesion molecule (CEACAM) (76, 77). TIM-3 functions as a direct negative regulator of T cells. Interaction with its various ligands results in T cell exhaustion as well as expansion of myeloid-derived suppressor cells (MDSCs) in the TME creating favorable conditions for tumor growth. Not surprisingly, TIM-3 levels have been found elevated in several malignancies. Blockade of TIM-3 decreases MDSCs while increasing T cell proliferation and cytokine production leading to anti-tumor activity (78). However, there has been some concern over TIM-3 blockade. Considering its role in immune responses against listeria and mycobacteria, inhibiting TIM-3 may result in an increased risk of these infections (79). Nevertheless, antibodies targeting this receptor have proceeded to clinical trials.

T cell immunoglobulin and ITIM domain (TIGIT) is a receptor part of the CD28 family and is expressed by T cells and natural killer cells (80). CD155 and CD112 are ligands that interact with TIGIT to bring about immunosuppressive effects (81). Studies have shown that tumor-infiltrating lymphocytes have elevated levels of TIGIT co-expressed with PD-1, LAG-3 and TIM-3 suggesting a role in tumor progression. Dual blockade of TIGIT and either TIM-3 or PD-1 has revealed an anti-tumor mechanism through immune cell proliferation, cytokine release and reversal of T cell exhaustion (82).

V-domain Ig suppressor of T cell activation (VISTA) is an unusual immune checkpoint with dual function as an inhibitory and stimulatory molecule (83). VISTA, expressed as a receptor on T cells, interacts with VSIG-3 on tumor cells to suppress T cell activation, proliferation and production of cytokines promoting tumor progression. This co-inhibitory pathway therefore presented as an alternative strategy for blockade therapy (84). Although most studies have described the inhibitory effects of VISTA on immune responses, other studies have demonstrated that VISTA can act as a ligand expressed on APCs allowing for immune activation. Regardless, blockade of VISTA seemed to enhance T cell infiltration and reduce myeloid suppressive cells proving to be an effective anti-tumor strategy (85, 86).

B7 homolog 3 (B7-H3) is a transmembrane protein found on various solid organs as well as immune cells such as APCs, T cells, B cells and natural killer cells. Although the exact ligand remains unknown, B7-H3 is believed to interact with the CD28 receptor family (87). This interaction prevents T cell activation, proliferation, cytokine production and appears to enhance cancer aggressiveness. B7-H3 blockade promotes T cell activation, cytokine release and cytotoxic activity. Moreover, it has been associated with fewer immune-related adverse events (irAEs) due to the lower expression of B7-H3 in normal tissues as opposed to the TME allowing for localised effects (88).

Drugs targeting LAG-3, TIM-3, TIGIT, VISTA and B7-H3 that are currently in clinical trials are listed in **Table 2**. Apart from these immune checkpoints, drugs associated with inhibitory targets beyond traditional immune checkpoints which lead to indirect repercussions on T-cell effect are also being investigated as next generation inhibitors. This has been reviewed in detail elsewhere (92).

**Table 2:** Next generation immune checkpoint inhibitors.

Target	Binding partner	Drugs	Trial stage	References
**LAG-3**	MHC-II	Eftilagimod alpha (Immutep)	I/II	(89)
		Relatimab (Bristol Myers Squibb)	II/III	
		Ieramilimab (Novartis)	II	
		Favezelimab (Merck)	I/II	
		Fianlimab (Regeneron)	I	
		Encelimab (AnaptysBio/GlaxoSmithKline)	I	
		Miptenalimab (Boehringer Ingelheim)	I	
		Sym 022 (Symphogen)	I	
		FS118 (F-star)	I	
		Tebotelimab (MacroGenics)	I	
**TIM-3**	Galactine-9, phosphatidyl serine, CEACAM	TSR-022 (GlaxoSmithKline)	I	(75)
		Sabatolimab (Novartis)	I/II	
		Sym 023 (Symphogen)	I	
		INCAGN 2390 (Incyte Corporation)	I	
		LY3321367 (Eli Lilly and Company)	I/II	
		BMS-986258 (Bristol Myers Squibb)	I/II	
		SHR-1702 (Jiangsu HengRui)	I	
**TIGIT**	CD155, CD112	Vibostolimab (Merck)	III	(80)
		Etigilimab (OncoMed Pharmaceuticals)	I	
		Tiragolumab (Genentech)	II	
		BMS-986207 (Bristol Myers Squibb)	I/II	
		Domvanalimab (Arcus Biosciences)	I	
**VISTA**	VSIG-3	JNJ-61610588 (Johnson & Johnson)	I	(90)
		CI-8993 (Curis Inc)		
**B7-H3**	Unknown	Enoblituzumab (MacroGenics)	II	(91)
		^131^I-omburtamab (Y-mAbs Therapeutics)	II/III	
		^124^I-omburtamab (Y-mAbs Therapeutics)	I	

### Challenges Associated With Immune Checkpoint Inhibitors

Immune checkpoint blockade (ICB) therapy has become one of the most successful cancer treatment strategies developed to date. A pooled meta-analysis study evaluating the long-term survival of 1861 advanced melanoma patients, receiving ipilimumab therapy, estimated a 3-year survival rate of 22% (93). The significance of these results is highlighted when compared to melanoma patients treated with dacarbazine, a chemotherapeutic agent, and 3-year survival rates were only 12.2% (14). In comparison to chemotherapeutics, ICB has allowed better disease control and outcomes for some patients. Accordingly, immunotherapy is now at the forefront for management of various malignancies. But despite the remarkable progress, ICB is challenged by low response rates, immune-related adverse events (irAEs) and resistance to treatment.

Response rates are known to vary depending on the type of malignancy. While excellent response rates are seen in Hodgkin’s lymphoma and melanomas which range from 40-70%, response rates in most other diseases is limited to only 10-25% (94). The unfortunate reality is that majority of patients do not experience any benefit from treatment with immune checkpoint inhibitors, and those that do, are likely to experience irAEs. Immune-related adverse events are caused from non-specific activation of the immune system resulting in immune responses that target self-antigens. ICB therapy most frequently results in dermatological irAEs such as pruritis and mucositis (68% of patients on ipilimumab therapy). Gastrointestinal distress and immune mediated colitis have also been reported in 40% of patients on ipilimumab therapy. Less common irAEs include endocrinopathies, hepatotoxicity, pneumonitis, renal toxicity, pancreatitis, neurotoxicity, cardiovascular toxicity and hematological abnormalities (95, 96). Inhibition of CTLA-4 has been associated with a higher frequency and severity of irAES than checkpoint inhibitors targeting the PD-1/PD-L1 axis (97). Although irAEs can be managed, they often lead to the discontinuation of treatment in some patients. Lastly, a crucial limitation of ICB therapy is related to resistance. Patients that fail to respond to treatment (innate resistance) and patients that respond initially but eventually develop disease progression (acquired resistance) will be discussed further.

### Mechanisms of Resistance to Immune Checkpoint Inhibitors

Immune checkpoint inhibitors targeting the CTLA-4, PD-1, and its ligand PD-L1 have been successful at inducing an anti-tumor immune response in several cancers (98). Ipilimumab was the first agent in the class of immune checkpoint inhibitors (ICIs) to be granted FDA approval for the treatment of metastatic melanoma in 2011 albeit with significant immune-related adverse events (irAEs) which needed to be addressed (99). Since then, diverse ICIs targeting the PD-1 (cemiplimab, nivolumab and pembrolizumab), and PD-L1 (atezolizumab, avelumab and durvalumab) have been granted FDA approval for the treatment of various cancers. To date there are several other ICIs currently in clinical trials. Although these agents have been successful at maintaining a sustained response in some cancer patients, the overall response is usually low, and some patients develop resistance over time (100). Resistance to ICIs may be innate (primary) or acquired (secondary). Resistance can also be classified as intrinsic or extrinsic to tumors. In intrinsic resistance, tumor cells modify processes associated with DNA damage response, cell signalling pathways and immune recognition. Extrinsic resistance occurs external to tumor cells and is facilitated by interactions of immune cells and non-immunological mechanisms in the tumor microenvironment (101–105).

Successful blockade of CTLA-4 and PD-1/PD-L1 in tumors results in reactivation and proliferation of T-cells. Activation of T-cells is dependent on the successful presentation of tumor antigens by APCs and the recognition of these antigens by MHC I and/or II. T-cells recognise the MHC-bound antigens and stimulate T-cell proliferation through co-stimulatory factors described previously (106). Both CTLA-4 and PD-1/PD-L1 pathways play a significant role in tumor evasion through down regulation of the immune response. Tumors evolve mechanisms to evade immune checkpoint blockade, thereby reducing the effectiveness of ICI therapy. In the following sections, we describe the various mechanisms that govern the evasion of T cell cytotoxicity by tumor cells following treatment with ICIs.

## Innate and Acquired Resistance

### Tumor Neoantigens

Innate or primary resistance is observed in tumors that have never responded to the initial treatment with ICIs (104). The most notable trigger of intrinsic resistance relates to genetic and epigenetic alterations that influence tumor neoantigen presentation, structure, and processing (106, 107). Neoantigens are peptides produced in the tumor because of somatic mutations that occur in cancer cells (108). The tumor neoantigen repertoire is crucial for the activation of an immune response and recruitment of effector T-cells to the tumor. Tumors with high mutational rates are typically responsive to ICI therapy compared to tumors with low tumor mutational burden (TMB) apart from renal cell carcinomas (66, 109–111).

Emerging evidence indicates that some tumors lose or down regulate generation of neoantigens required to illicit an immune response and therefore the tumor escapes T-cell cytotoxicity (63). Anagnostou and colleagues (63, 82) assessed biopsies of relapsed NSLC patients and observed a downregulation of key tumor antigens indicative of an anti-PD-1 and anti-CTLA-4 resistance. Efficacy of anti-PD-1 inhibitors is dependent on the availability of tumor antigen specific T-cells in the tumor microenvironment and the upregulation of PD-1 in effector T-cells and PD-L1 in tumor cells. This requires tumors to present specific antigens that are different from the original tumor cells. Without these antigens, the immune checkpoint blockade is attenuated.

In addition to tumor neoantigen downregulation, tumors escape immunosuppression through alteration of the antigen presentation machinery. Dendritic cells (DCs) initiate an immune response through uptake and presentation of tumor antigens to activate naïve CD4 and CD8 T cells (112, 113). DCs activate the CD8 T cells in a process called cross priming where antigens are presented to CD8 T cells *via* MCH I to generate an anti-tumor CD8 T cell response (114). Cross priming of tumor specific CD8 T cells is very important in initiation and stabilisation of the anti tumor immune response. Deficiencies in T cell priming mechanism have been shown to contribute unresponsiveness to immune checkpoint inhibition therapy (115). The TME plays a major role in the transportation of effector CD8 T cells to tumors and alterations in the TME therefore affect the anti-tumor response. In particular, the presence of tumor derived inhibitory molecules such as interleukin (IL-6, 10), transforming growth factor beta (TGFβ) and VEGF produced by the tumor negatively impact the growth, maturation and differentiation of DCs (116, 117). These molecules are usually secreted by myeloid derived suppressor cells (MDSCs), tumor-associated macrophages (TAMs) and regulator T cells (Tregs) which are discussed in subsequent sections.

### Dysfunctional Major Histocompatibility Complex Molecules

Alterations in the structure of MHC-I/II and the antigen presenting machinery, beta 2 microglobulin (B2M), prevents the identification and presentation of tumor antigens (118). The MHC class I pathway is responsible for antigen presentation and any defects in the genes associated with MHC-1 pathways such as the HLA class I and the *B2M* gene affect antigen presentation and ultimately immune response (119). This phenomenon has been observed in several tumors with *B2M* mutations and more specifically the loss of heterozygosity (LOH) of the *B2M* gene. Indeed, these modifications have been observed in various tumor tissues and have been associated with resistance to anti-PD-1/PD-L1 and anti-CTLA-4 immune checkpoint inhibitors (120–122).

### Inadequate Anti-Tumor T-Cell Effector Function

Interestingly, mutations in the JAK1 and JAK2 pathways have also been associated with resistance to ICI treatment (123, 124). JAK1/2 are key intermediates in the interferon signaling pathways. Since the interferon pathway (INF) is particularly involved in the upregulation of PD-L1 expression, blockade of the PD-1/PD-L1 is likely ineffective in tumors with alterations in the interferon pathway. This is suggestive of an alternate mechanism of immune evasion in tumor cells other than PD-1/PD-L1 upregulation (125). Moreover, Gao and colleagues reported anti-CTLA-4 resistance in tumors with LOH in many genes associated with the INFγ pathway (126). It has been shown in melanoma that the interferon-gamma-JAK1/JAK2-STAT1/STAT2/STAT3-IRF1 signaling cassettes primarily regulates PD-L1 expression on the cancer cell, through IRF1 binding to its promoter. This establishes PD-L1 as an interferon-γ immediate response gene. Upon tumor antigen recognition in the context of the MHC, the T cell releases interferon gamma that binds to its receptors on the cancer cell. This is followed by the transduction of a signal *via* the JAK/STAT pathway, culminating in the activation of the paralogous the PD-L1 and PD-L2 genes of the tumor cell (**Figure 2**). In this way interferon gamma can play a critical role in negative regulation of T cell activation through the expression of PD-1 receptors on the tumor cell. Immune checkpoint blockade therapy acts by blocking PD-1/PD-L1/2 interaction thereby restoring T cell activation and anti-tumor activity. The evidence shows that dysregulation of this pathway in the tumor cell produces resistance to PD-1 based ICB therapy. It was shown that loss of function mutations in Ak1/2 and subsequent lack of PD-L1 expression led to primary resistance to anti-PD-1 antibody therapy (123, 127). Similar interferon signalling dependent resistance has been demonstrated with the anti-CTLA-4 therapy, ipilimumab (126). Though studies on delayed relapses after anti-PD-1 therapy, the interferon-γ signalling pathway has been shown to be associated with acquired immunity to anti-PD-1 immune blockade therapy (124). The inhibitory CTLA-4 is essentially an intracellular molecule whose trafficking from intracellular vesicles to the to the cell surface is tightly regulated to maintain an optimal balance with stimulatory molecules (41).

### T-Cell Exhaustion

T cell exhaustion is a phenomenon that was first described in mice with chronic viral infections, and thereafter observed in humans with chronic viral infections and cancer (42, 128–131). More recently, however, it has been linked to resistance in ICB therapy. Exhausted T cells in the tumor microenvironment have been shown to progressively lose their functional capacity to proliferate, produce effector cytokines and lyse upon chronic antigen exposure (130, 131). While numerous pathways may individually influence T cell exhaustion, the PD-1/PD-L1 checkpoint pathway partly contributes to T cell exhaustion. In exhausted T cells, PD-1 expression is driven by demethylation of its promoter. The stability of this epigenetic mechanism blocks long-term effector function or memory development by T cells following ICB therapy, potentially explaining disease relapse in patients treated with PD-1/PD-L1 checkpoint inhibitors (132–134). Moreover, studies have reported that T cell exhaustion in acquired resistance is a consequence of the up-regulation of other checkpoint inhibitors such as TIM3, LAG3 and VISTA following checkpoint blockade (120, 135, 136). The exact mechanisms leading to T cell exhaustion following ICB therapy is largely unclear and further studies are required to validate the dysfunctional T cell states and their contribution to resistance.

### The Tumor Microenvironment (TME)

The TME contains various types of cells that play a significant role in the promotion or inhibition of the tumor. The cell types include regulatory T-cells (Treg cells), myeloid derived suppressor cells (MDSCs), cancer-associated adipocytes, fibroblasts and endothelial cells; and tumor-associated macrophages (TAMs) (137). Through producing various molecules, Tregs, MDSCs, TAMs and tumor-associated stromal cells inhibit the anti-tumor T-cell response and maintain an immune tolerant tumor that attenuates the effectiveness of ICIs (138, 139). Foxp3 Treg cells, are mainly produced by the thymus as a functionally mature and distinct T-cell subpopulation, whose function is to maintain self-tolerance after an immunological response or activation (140).

Treg cells produce immunosuppressive molecules including transforming growth factor-β (TGF-β) and interleukin-10 (IL-10) which typically interfere with the activation, proliferation and survival of effector T-cells (141). Additionally, Tregs also upregulate the expression of immune checkpoints such as CTLA-4, PD-1 and others (142). The effectiveness of anti-CTLA-4 mAb is dependent on decreasing Treg cells in tumors *via* antibody-dependent cytotoxicity but this mechanism does not affect the activation of CTLA-4 (143). For this reason, anti-CTLA-4 alone selectively depletes Treg cells permitting immunosuppression stimulated by remaining Treg cells (138, 144). Several animal studies have shown a connection between amount of Treg cells in the TME and enhanced antitumor immunity (145, 146). Studies in cancer patients treated with anti-CTLA-4 therapy revealed better response to treatment in patients with a low ratio of Treg cells compared to Teff cells in the TME (140, 147). Recruitment of Tregs in the TME relies upon metabolic processes associated with lipid metabolism. A study by Pacella and colleagues (2018) showed that both increased glycose and oxidative metabolism influenced Tregs expansion by fueling fatty acid (FA) synthesis (148).

Tumor-associated macrophages (TAMs) support tumor growth through the expression of PD-L1 ligand and Na/H exchanger isoform 1 (NHE1) (149, 150). NHE1 maintains the alkaline intracellular pH of glioma cells, a driving force of glycolytic metabolism exploited by cancer cells in a process called Warburg Effect (151, 152). Moreover, TAMs are involved in the production of cytokines such as transforming growth factor (TGF-β) and vascular growth factor (VEGF-A) implicated in tumor evasion (153, 154). Since TAMs can regulate the production of pro-inflammatory and immune response inhibitory molecules, anti-PD-L1 inhibition alone is not sufficient for prolonged suppression of the tumor.

Myeloid-derived suppressor cells (MDSCs) alter the function of CD8+ T cells through numerous mechanisms including a (i) decrease in arginine and cysteine production in the TME, (ii) reduced transport of T cells into the lymph node and tumor, (iii) production of free radicals that ultimately block TCR and IL-2 signaling, inducing T cell death and expansion of Tregs (155). Like TAMs, MDSCs may be induced by tumor-derived factors such as TGF-β, ILs 1, 6, 10 and VEGF-A. MDSCs cells have been shown to also express immune checkpoint PD-L1, further contributing to immunosuppression in mice models (156). The manifestation of MDSCs was associated with poor prognosis in metastatic melanoma patients treated with anti-CTLA-4 (ipillimumab) (157, 158).

### Metabolic Reprogramming in the TME

Cancer cells tend to accumulate metabolic alterations that allow them to utilize eccentric sources of nutrients to support cancer cell proliferation and deprive antitumor immune cells of nutrients within the tumor microenvironment. Because tumors are heterogeneous in nature, they often have complex metabolic patterns. The first evidence of variations in nutrient metabolism observed in cancer and normal cells was reported in the 1920s by Warburg and colleagues (159). They observed a marked increase in glucose metabolism in cancer cells compared to non-proliferating normal cells; and the preference of glycolysis over oxidative phosphorylation (OXPHOS) even in the presence of oxygen and functional mitochondria. The observed phenomenon was later termed the “Warburg Effect” (159). This observation was further corroborated in a variety of tumors associated with poor prognosis (160). Even though there are other metabolic processes and molecules governing tumor resistance, we will focus on the metabolism of glucose in the TME and its impact on tumor progression and antitumor immune escape.

The high demand for glucose in cancer cells within the TME starves immune cells resulting in poor antitumor immune response (51). When T cells are inactive, they largely rely on OXPHOS and fatty acid oxidation (FAO) to support their needs. Once T cells are activated through binding of costimulatory receptors such as CD28, T cells alter their metabolism to support T-cell proliferation and T cell effector (T_eff_) functions (161). The CD28 co-stimulation drives the activation of the PI3K/AKT pathways and glycolytic flux (162, 163). The dramatic increase in glycolysis in T cells is essential for T-cell growth, division, and differentiation into cytotoxic T cells (164). Since glucose is required by tumors and is essential to support immune cell growth, differentiation and function, its metabolism within the TME affects the function of immune cells infiltrating the TME (165). The competition for glucose metabolism within the TME deprives tumor infiltrating lymphocytes (TIL) of glucose resulting in their exhaustion and tumor immune escape (166).

In addition, the preference for aerobic glycolysis in tumors increases the levels of lactic acid in the TME resulting in an acidic environment that further supports the growth of tumors whilst inhibiting immune cell function within the TME. Indeed Muller and colleagues (2000) showed that the activation and function of tumor infiltrating immune cells (IL-2) was significantly perturbed in acidic conditions. Both the stimulated and unstimulated human PMBCs were unable to kill tumor cells after three days of culture in an acidic culture environment of pH 6.5 (167). This finding was further supported by Calcinotto and colleagues (168) using mice models and human tumor cell lines. Using *in vitro* and *in vivo* models, Calcinotto and colleagues revealed that the acidic microenvironment not only affected the function of effector cells but also induction of T-cell anergy (168). In addition to interfering with immune cell activation and function, acidic pH in the microenvironment also upregulates the expression of CTLA-4 on T lymphocytes, therefore intensifying antitumor resistance (169).

Besides the increased uptake of glucose by tumor cells; competitive uptake of other metabolites, amino acids (glutamine, arginine, tryptophan) and growth factors by tumor cells also affects the function of immune cells (51, 165).

Amino acids are protein building blocks, the high availability of amino acids in the TME is essential for tumor growth. At the same time, amino acids are essential for immune cells differentiation and development of their antitumor effector cells (170). For example, glutamine powers the tricarboxylic acid (TCA) cycle *via* glutaminolysis, to provide metabolic intermediates that serve as building blocks for lipids, proteins, and nucleic acids, which are necessary for cancer cell proliferation. Interestingly, the metabolic pathway used by the tumors has been shown to be essential for T cell activation and proliferation (171, 172).

There are several studies that have investigated the impact of targeting different metabolic pathways to assist the immune checkpoint inhibition or circumvent resistance. The metabolic dependencies between tumor and immune cells in the TME make it challenging to obtain antitumor effects with drugs targeting metabolic processes (170). Targeting enhanced glycolytic activity of tumors through inhibition of glycolysis regulatory enzymes or *via* application of competitive glucose analogs has been shown to promote T- cell proliferation and function (166, 173). Various studies have shown that the blockade of immune checkpoints (PD/PD-L1 and CTLA-4) rescues TILs from tumor-induced glucose restrictions and restores glycolysis in T-cells.

## Strategies to Overcome Resistance to Immune Checkpoint Blockade Therapy

When looking at patients that experience resistance, it is helpful to define them into two broad categories, firstly are the ones with innate resistance, who never respond to the immune checkpoint therapy (ICT) and secondly are the ones who have acquired resistance, who respond positively to treatment at first, but then build up a resistance resulting in the treatment becoming ineffective over time (102). Studies have found that tumors that are infiltrated by T cells and therefore that have initiated an inflammatory response as well as have a higher mutational burden have a better response to ICT then tumors that do not, this is especially important when looking at potential strategies to combat resistance to ICI (174).

An essential aspect to combating ICT resistance requires a deeper understanding of the exact mechanisms involved, down to an individual level, so that therapies can be adapted to the tumor microenvironment. To overcome resistance against a single checkpoint inhibitor target, combinational therapies have been conducted. Multiple combinations of different therapies have been successfully tried with the most promising combination therapies including ICT paired with (i) other checkpoint inhibitors, with a combination of anti-PD-1 and anti-CTLA-4 already having been approved for multiple cancers as they have been shown to improve T-cell activation and decrease T-cell exhaustion (175) and combinations with next generation ICT such as anti-LAG and anti-TIGHT showing similar positive results (176). (ii) Immunotherapeutic agents such as cancer vaccines and oncolytic virus therapy which can improve antigen presentation and recognition and T cell infiltration (177, 178). (iii) Removal of co-inhibitory signals and activation of co-stimulatory signals which can amplify T cell activation and T cell cytotoxicity (179). (iv) DNA damaging therapies such as chemotherapy or radiation which has been seen to increase antigen presentation, pro-inflammatory cytokines and activation of dendritic cells, to stimulate the presentation of neoantigens in non-inflamed, non T cell infiltrated tumor cells (180) and (v) more targeted therapies including monoclonal antibodies and tyrosine kinase inhibitors which have been seen to enhance antitumor immunity, increase T cell infiltration and decrease T cell exhaustion (181).

In addition to these, epigenetic modifications within cancer cell DNA can impact the presentation and processing of antigens, which can promote immune evasion, therefore, demethylating agents may also increase the response to combination ICT treatment as they have been seen to elicit an immunostimulatory response, upregulation of cytokine production as well antigen presentation and inhibition of T regulation cells (182). Interestingly a link has been reported between the gut microbiome and response to ICT, where mice suffering from sarcomas that were fed with a germ-free diet, had a very poor response to CTLA-4 blockade therapy. This was further supported when their response was restored upon being fed with *Bacteroides fragilis* (183). This has since been concluded in a number of studies that demonstrate that gut microbiome can affect a person’s response to ICT treatment (184).

Lastly biomarkers have also become a topic of interest in helping overcome resistance as they can be investigated to estimate the predicted response of an individual to treatment. Biomarkers of particular interest include PD-L1 expression, tumor mutation burden (TMB), microsatellite instability-high (MSI-H) or mismatch repair (MMR) deficiency, IFN-γ signalling and T-cell infiltration (185). The only predictive biomarker that has been approved to date is PD-L1 expression using immunohistochemistry (IHC), in which higher expression correlates to a positive response to ICT and fewer side effects observed (186). However, because the detection of PD-L1 relies on antibody staining techniques, this creates inconsistencies in the accuracy of results and therefore its predictive value (187). TMB as a potential biomarker looks for somatic mutations *via* DNA sequencing, with an increased number of mutations resulting in higher neoantigen production and therefore a positive response to ICT, however not all mutations and neoantigens correlate equally towards a positive response (188). Defective DNA mismatch repair (MMR) can lead to high microsatellite instability (MSI-H), and MSI-H is associated with higher neoantigen production by tumors and therefore a stronger immune response and better response to ICT. MSI has been argued to be the most accurate biomarker predictor (189).

Activation of IFN-γ signalling can be used as a predictive biomarker as studies have found loss of function mutations or gene knockdowns in this pathway result in resistance to ICT treatment (7, 23, 182). IFN-γ signalling up regulates the major histocompatibility complex II as well as antigen presenting cells (APCs) and increases PD-L1 expression, however on the other hand studies have found that chronic IFN-γ signalling can lead to acquired resistance, therefore it seems early IFN-γ signalling may predict positive response to ICT but once resistance is acquired, continued IFN-γ signalling can predict further resistance (190). Lastly decreased T cell infiltration and a lack of an inflammatory response has been reported to be linked to poorer prognosis and is therefore predictive of a low response to ICT (191).

## Future Directions

This review examined the successes and failures of immune checkpoint inhibitors (ICIs) and focused on resistance mechanisms. Although ICIs have produced unmatched and durable clinical responses in some cases, this revolutionary strategy has not succeeded in most patients. The limited application of this revolutionary cancer treatment strategy is the most critical matter and is a subject of intense investigation. Critically, it is not possible to predict who is likely or unlikely to benefit from ICI therapy. Towards this end, the discovery of biomarkers is ongoing and is expected to allow personalized treatment approaches. Also, the immune-related adverse effects present a difficult challenge because they are unique and unlike adverse effects often seen with traditional treatments. Although irAEs are usually low-grade and reversible, they can also cause permanent disorders and affect any organ. Another challenge to ICI treatment is that poorly understood primary or secondary resistance limits treatment outcomes. The enormous impact of the tumor microenvironment on carcinogens adds a chaotic dimension to the study of cancer as the TME is a dynamic system and pliable. Presumably, there are deterministic laws or logical patterns that govern the apparent random environment. With the advances in artificial intelligence and high-throughput data, it is possible to produce knowledge to understand the complex emergence of irAEs better. The ongoing transcriptomic and epigenetic analyses are likely to make invaluable knowledge in this regard.

## Conclusion

Immune checkpoint therapy (ICT) is a very promising, recently developed cancer treatment. Here, we described PD-1/PD-L1 and CLTA-4 immune checkpoints and the monoclonal antibody drug inhibitors that have been approved by the FDA. Although there are positive results in some patients treated with immune checkpoint inhibitors, others never respond to treatment, while the responders often develop resistance. We have described various mechanisms by which resistance can develop and some efforts to overcome this problem. The diverse components of the tumor microenvironment play a critical role in creating ICT resistance. Strategies currently used to help combat resistance include combination therapy with multiple checkpoint inhibitors or checkpoint inhibitors with chemotherapy or radiation.

Given the increasing incidence of cancer, there is an urgent need to improve the currently available therapies and develop new alternatives. Although glucose competition exerts pressure on normal cells in the tumor microenvironment, the fine details about how this affects ICI therapy is still unclear.

## Author Contributions

BLR, SAS and STM contributed equally in the, composition of the main text. LD contributed in the conception of article, in intellectual input and in fundraising. MN is the corresponding author who researched and wrote the final document. All authors contributed to the article and approved the submitted version.

## Funding

BLR and LD are funded by The Technology and Human Resources for Industry programme (THRIP). SAS and STM are funded by the National Research Foundation (NRF) GUN: 116681 and GUN: 121878 respectively.

## Conflict of Interest

Authors BLR and LJD were employed by company Buboo (Pty) Ltd.

The remaining authors declare that the research was conducted in the absence of any commercial or financial relationships that could be construed as a potential conflict of interest.
